# Trends in medicare reimbursement for the resection of spinal tumors: 2000–2021

**DOI:** 10.1007/s10143-025-03742-4

**Published:** 2025-08-19

**Authors:** Shravan Atluri, Dylan Dupont, Spencer Singh, Christiana Cornea, Rebecca M. Garner, Sanjit Shah, Joseph Cheng, Rani Nasser

**Affiliations:** 1 Idaho College of Osteopathic Medicine, 1401 E Central Dr, Meridian, ID 83642 USA; 2 Department of Neurosurgery, University of Cincinnati College of Medicine, 3230 Eden Ave, Cincinnati, OH 45267 USA

**Keywords:** Medicare, Reimbursement, Spine, Current procedural terminology, CPT code

## Abstract

Despite recent fiscal expansion and rising healthcare expenditures, reimbursement rates for specific surgical procedures like spinal tumor resections remain underexplored. This study analyzes trends in real-value Medicare Part B reimbursements for spinal tumor resections over a 21-year period by querying the Medicare Part B database using relevant CPT codes from 2000 to 2021. Data included the aggregate number of procedures, allowed charges, and payments per procedure. Average payments per procedure and expected patient payments were calculated based on the 20% coinsurance of Medicare Part B. Compound Annual Growth Rates (CAGR) were determined and adjusted for inflation using the Consumer Price Index for All Urban Consumers (CPI-U) and the Medical Care component (CPI-MEDSL), representing out-of-pocket expenses. Generally speaking, the volume of spinal tumor surgeries increased by 28.77%, and average gross reimbursement rose by 13.57% without inflation adjustment. After adjusting for inflation, average reimbursement decreased by 27.83%. The CAGR analysis showed a 1.23% annual increase in procedures and a 0.64% annual increase in unadjusted reimbursements, but a 1.66% annual decrease when adjusted for inflation. Patient payments increased by 31.55% (CAGR of 1.31%); however, when adjusted for the 201.4% rise in medical care costs, patient payments effectively declined by 44.63%, with an annual decrease of 2.92%. Despite increased nominal healthcare spending, inflation-adjusted reimbursement rates for spinal tumor resections have declined. This trend raises concerns regarding the sustainability of current reimbursement policies and their impact on maintaining advancements in spinal surgery.

## Introduction

Physician responsiveness to the pressures of surrounding incentives has been well documented, with a positive correlation between physician behavior and payment [1–3]. Therefore, the dynamics of healthcare reimbursement represent a meaningful aspect of clinical practice and healthcare policy. This study aims to provide a comprehensive analysis of the trends in Medicare reimbursement for the resection of spinal cord tumors over a twenty-one-year period, from 2000 to 2021. While similar analyses concerning Medicare reimbursement for surgeries targeting non-neoplastic spinal lesions have been performed, there is a dearth of literature regarding Medicare reimbursement for resection of spinal tumors [4, 5]. Therefore, the rationale for focusing on this specific timeframe and category of surgical interventions is twofold: first, to understand the financial trajectory for a necessary subset of intricate and resource-intensive neurosurgical interventions, and second, to further contextualize these changes within the shifting landscape of healthcare economics and policy.

Medicare Part B, the largest payor in the United States healthcare system, influences not only federally funded health insurance programs but also sets a precedent for private insurance payors. Medicare Part B provides coverage for those aged 65 years and older, with 95% of those eligible enrolled for coverage. The issue arises in this group's rise in population due to the aging of the baby boomer cohort, the largest current population cohort in the US. This is expected to be a significant financial burden due to the strong correlation between aging and infirmities, such as cancer [6–10] leading to a rise in aggregate spending as an expected consequence of their progressively greater need for medical care. It is also likely a sign that Medicare will continue making in-roads as a principal healthcare payor in spaces of surgical intervention such as spinal tumor resection. Additionally, as technological advancements are likely to continually increase requisite capital expenditures for sophisticated spinal surgeries, continuous assessments of Medicare’s efficacy as a principal payor are necessary [11, 12]. If reimbursements lag behind the ever-rising demands, costs, and complexities of these procedures, it may be possible that our payment systems could inadvertently stifle future innovation and quality of care.

In examining these trends, we aim to contribute to the ongoing discourse on the economic aspects of neurosurgical care, offering insights that are critical for policy makers, healthcare providers, and stakeholders in the neurosurgical field. This study endeavors to provide insight into the financial underpinnings of spinal cord and vertebral column tumor resections. The present observations may inform strategies for sustainable practice management and equitable reimbursement advocacy that will help to ensure continued advancement and accessibility of high-quality neurosurgical care.

## Methods

### Data acquisition and organization

The study utilized reimbursement data spanning from the year 2000 to 2021, sourced from the Medicare Part B National Summary data files available on the CMS. The data was organized using Current Procedural Terminology (CPT) codes that were directly related to the excision of spinal tumors within the vertebral column as well as within the spinal cord, and then organized into groups based on tumor location and associated surgical procedure. The groups were numbered 1–7 as follows:

1. Laminectomy for biopsy/excision of intraspinal neoplasm; extradural. This group included CPT codes 63275, 63276, 63277, 63278.
2. Laminectomy for biopsy/excision of intraspinal neoplasm; intradural, extramedullary. This group included CPT codes 63280, 63281, 63282, 63283.
3. Laminectomy for biopsy/excision of intraspinal neoplasm intradural, intramedullary + the combination intradural/extradural code. This group included CPT codes 63285, 63286, 63287, 63290.
4. Vertebral Corpectomy (vertebral body resection), partial or complete for excision of intraspinal lesion, single segment, extradural. This group included CPT codes 63300, 63301, 63303.
5. Vertebral Corpectomy (vertebral body resection), partial or complete for excision of intraspinal lesion, single segment, intradural. This group included CPT codes 63304, 63305, 63306, 63307.
  - This coding group was affected by incomplete data with the allowed services not being recorded for several years during the timeframe of interest, as a result it was excluded from overall statistics.
6. Partial excision of posterior vertebral component for intrinsic bony lesion, single vertebral segment. This group included CPT codes 22100, 22101, 22102.
7. Partial excision of vertebral body, for intrinsic bony lesion, without decompression of spinal cord or nerve roots without decompression of spinal cord or nerves. This group included CPT codes 22110, 22112, 22114.

The data extracted involved primarily three measures for each CPT code.

1. Allowed Services: Total number of specific medical services, procedures, or items that Medicare has approved for reimbursement within a given time frame.
2. Allowed Charges: The total charge amount accepted for reimbursement by Medicare Part B
3. Payment: The amount that is reimbursed by Medicare Part B, representing typically 80% of the allowed charges, with the remaining 20% often paid for via coinsurance.

### Data analysis

All CPT codes were analyzed along the parameters of interest as described below. No analyses were performed to assess relationships between categorical variables (i.e., included CPT codes) Detailed financial analysis was conducted involving the following three primary aspects:

1. Assessment of Reimbursement Rates: The raw percentage change and the average reimbursement rates were evaluated for each procedure. This assessment offered insights into the financial trends and patterns over the two-decade span. With the separation of allowed charges and payments, we were additionally able to derive the average patient payment for each procedure, which is in-line with the 20% coinsurance that is part of Medicare Part B.
2. Inflation Adjustment: All financial data were adjusted for inflation. This adjustment utilized the consumer price index for all urban customers (CPI-U) as reported by the Bureau of Labor Statistics (BLS) and the Federal Reserve Economic Data (FRED) [13] as a standard measure, ensuring that the financial analysis reflected real value changes rather than nominal fluctuations.
3. Medical Costs Inflation Adjustment: For the patient payments, the CPI for all urban consumers: medical care in U.S city average (CPI-MEDSL) was utilized, which is a representation of all out-of-pocket costs for medical care for the average urban consumer. This comparison allowed us to analyze the trend for spinal tumor resections directly compared to the average patients’ medical expenses over the same time period.

Additionally, we determined the growth rates associated with each procedure. This analysis encompassed:

1. Average Annual Percentage Change: Yearly percentage change was calculated to provide insights into the short-term trends in reimbursement rates.
2. Total Percentage Change: The cumulative change over the entire period of study was assessed to understand the long-term trends.
3. Compound Annual Growth Rate (CAGR): This metric provides an average growth rate over multiple periods, evening out the variability between yearly rates and offering a view of long-term trends. The CAGR equation employed is as follows:
  $$CAGR = (\frac{2021\;Medicare\;Reimbursement}{2000\;Medicare\;Reimbursement}{)}^{1/(2021-2000)}-1$$

Finally, utilizing the data collected, trend analysis was performed for all included procedures and their associated pricing data which was then subsequently repeated following price correction. Both average annual and total percentage change were calculated based on these trends. Pearson correlation coefficients were then calculated to quantify the linear relationship between CPI and both average nominal and inflation-adjusted Medicare payments. The statistical significance of these correlations was then assessed using *p*-values.

## Results

### Group 1- Laminectomy for intraspinal neoplasm; extradural (Fig. 1a, b and c)

**Fig. 1:**
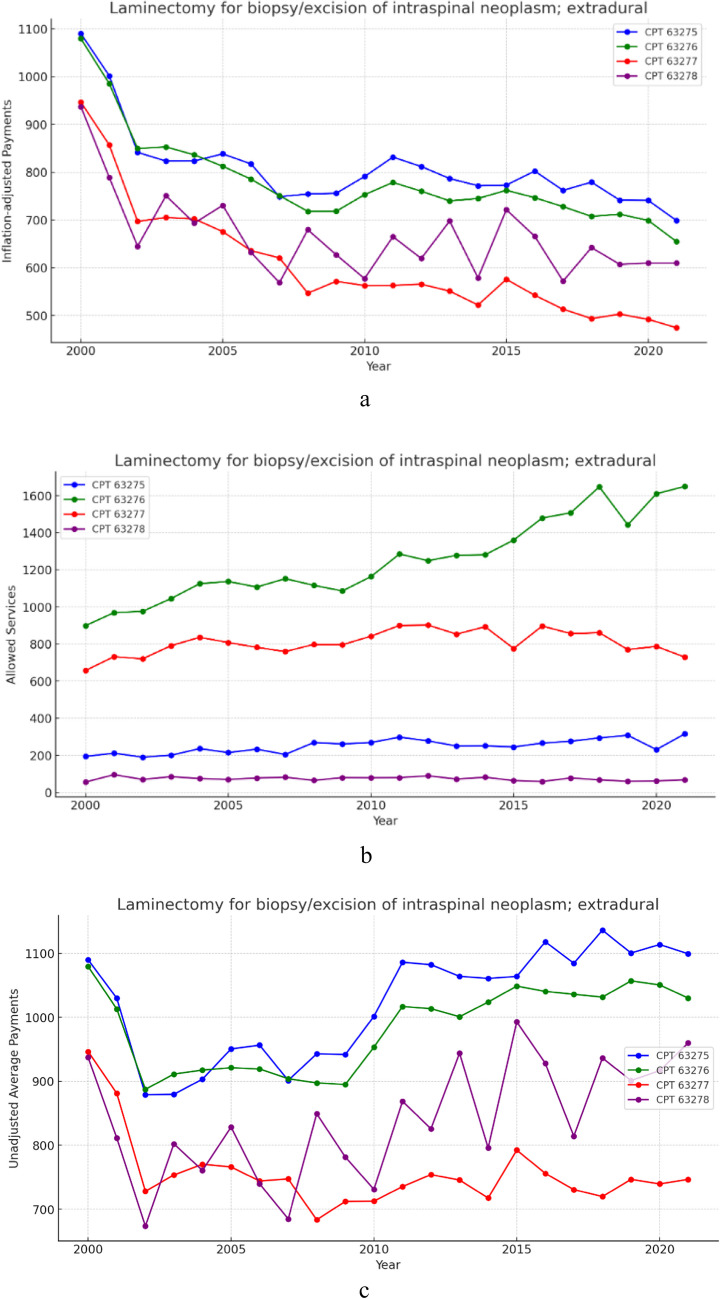
**a** 21 year, inflation adjusted payment trends for the selected procedure codes within Laminectomy for biopsy/excision of intraspinal neoplasm; extradural. **b** 21 year, allowed services trends for the selected procedure codes within laminectomy for biopsy/excision of intraspinal neoplasm; extradural. **c** 21 year, unadjusted average payment trends for the selected procedure codes within laminectomy for biopsy/excision of intraspinal neoplasm; extradural

From 2000–2021, allowed services for procedures within the extradural group increased on average by 44.68% (Table 1). Meanwhile, average unadjusted payment decreased by 5.60% (Table 2) and inflation adjusted payment on average declined in real-value by 40.01% (Table 3). Results from the CAGR analysis indicate that, across this group, allowed services increased by an average of 1.68% year over year (Table 1). However, unadjusted payments decreased by an average of 0.30% annually (Table 2), and inflation-adjusted payments fell by 2.43% annually (Table 3).

**Table 1:** Total change and CAGR for all included allowed services form 2000–2021

Grouping	CPT Code	Procedure Description	Total Change in Allowed Services (%)	CAGR
Group 1	63275	Laminectomy for biopsy/excision of intraspinal neoplasm; extradural, cervical	62.89%	2.35%
	63276	Laminectomy for biopsy/excision of intraspinal neoplasm; extradural, thoracic	83.43%	2.93%
	63277	Laminectomy for biopsy/excision of intraspinal neoplasm; extradural, lumbar	10.96%	0.50%
	63278	Laminectomy for biopsy/excision of intraspinal neoplasm; extradural, sacral	21.43%	0.93%
Group 1 Average			44.68%	1.68%
Group 2	63280	Laminectomy for biopsy/excision of intraspinal neoplasm; intradural, extramedullary, cervical	9.50%	0.43%
	63281	Laminectomy for biopsy/excision of intraspinal neoplasm; intradural, extramedullary, thoracic	11.95%	0.54%
	63282	Laminectomy for biopsy/excision of intraspinal neoplasm; intradural, extramedullary, lumbar	1.36%	0.06%
	63283	Laminectomy for biopsy/excision of intraspinal neoplasm; intradural, sacral	0.00%	0.00%
Group 2 Average			5.70%	0.26%
Group 3	63285	Laminectomy for biopsy/excision of intraspinal neoplasm; intradural, intramedullary, cervical	−23.81%	−1.05%
	63286	Laminectomy for biopsy/excision of intraspinal neoplasm; intradural, intramedullary, thoracic	2.78%	0.12%
	63287	Laminectomy for biopsy/excision of intraspinal neoplasm; intradural, intramedullary, thoracolumbar	−29.85%	−2.05%
	63290	Laminectomy for biopsy/excision of intraspinal neoplasm; combined extradural-intradural lesion, any level	−22.86%	−1.05%
Group 3 Average			−18.44%	−1.01%
Group 4	63300	Vertebral corpectomy (vertebral body resection), partial or complete, for excision of intraspinal lesion, single segment; extradural, cervical	37.30%	2.23%
	63301	Vertebral corpectomy (vertebral body resection), partial or complete, for excision of intraspinal lesion, single segment; extradural, thoracic by thoracolumbar approach	28.05%	1.29%
	63303	Vertebral corpectomy (vertebral body resection), partial or complete, for excision of intraspinal lesion, single segment; extradural, lumbar or sacral by transperitoneal or retroperitoneal approach	62.90%	2.90%
Group 4 Average			42.75%	2.14%
Group 5	63304	Vertebral corpectomy (vertebral body resection), partial or complete, for excision of intraspinal lesion, single segment; intradural, cervical	−33.33%	−2.00%
	63305	Vertebral corpectomy (vertebral body resection), partial or complete, for excision of intraspinal lesion, single segment; intradural, thoracic by transthoracic approach	N/A	N/A
	63306	Vertebral corpectomy (vertebral body resection), partial or complete, for excision of intraspinal lesion, single segment; intradural, thoracic by thoracolumbar approach	N/A	N/A
	63307	Vertebral corpectomy (vertebral body resection), partial or complete, for excision of intraspinal lesion, single segment; intradural, lumbar or sacral by transperitoneal or retroperitoneal approach	N/A	N/A
Group 5 Average			N/A	N/A
Group 6	22100	Partial excision of posterior vertebral component (spinous process, lamina or facet) for intrinsic bony lesion, single vertebral segment; cervical	36.90%	2.08%
	22101	Partial excision of posterior vertebral component (spinous process, lamina or facet) for intrinsic bony lesion, single vertebral segment; thoracic	101.75%	4.57%
	22102	Partial excision of posterior vertebral component (spinous process, lamina or facet) for intrinsic bony lesion, single vertebral segment; lumbar	78.76%	3.73%
Group 6 Average			72.47%	3.46%
Group 7	22110	Partial excision of vertebral body, for intrinsic bony lesion, without decompression of spinal cord or nerve root(s), single vertebral segment; cervical	152.08%	6.90%
	22112	Partial excision of vertebral body, for intrinsic bony lesion, without decompression of spinal cord or nerve root(s), single vertebral segment; thoracic	−29.03%	−1.67%
	22114	Partial excision of vertebral body, for intrinsic bony lesion, without decompression of spinal cord or nerve root(s), single vertebral segment; lumbar	−46.77%	−2.67%
Group 7 Average			25.43%	0.85%
Total

**Table 2:** Total change and CAGR for nominal payments from 2000–2021

Grouping	CPT Code	Procedure Description	Total Change in Nominal Payment (%)	CAGR
Group 1	63275	Laminectomy for biopsy/excision of intraspinal neoplasm; extradural, cervical	0.88%	0.04%
	63276	Laminectomy for biopsy/excision of intraspinal neoplasm; extradural, thoracic	−4.56%	−0.22%
	63277	Laminectomy for biopsy/excision of intraspinal neoplasm; extradural, lumbar	−21.13%	−1.12%
	63278	Laminectomy for biopsy/excision of intraspinal neoplasm; extradural, sacral	2.41%	0.11%
Group 1 Average			−5.60%	−0.30%
Group 2	63280	Laminectomy for biopsy/excision of intraspinal neoplasm; intradural, extramedullary, cervical	8.47%	0.39%
	63281	Laminectomy for biopsy/excision of intraspinal neoplasm; intradural, extramedullary, thoracic	4.71%	0.22%
	63282	Laminectomy for biopsy/excision of intraspinal neoplasm; intradural, extramedullary, lumbar	1.81%	0.09%
	63283	Laminectomy for biopsy/excision of intraspinal neoplasm; intradural, sacral	−19.36%	−1.02%
Group 2 Average			−1.09%	−0.08%
Group 3	63285	Laminectomy for biopsy/excision of intraspinal neoplasm; intradural, intramedullary, cervical	4.02%	0.19%
	63286	Laminectomy for biopsy/excision of intraspinal neoplasm; intradural, intramedullary, thoracic	−4.76%	−0.22%
	63287	Laminectomy for biopsy/excision of intraspinal neoplasm; intradural, intramedullary, thoracolumbar	10.95%	0.47%
	63290	Laminectomy for biopsy/excision of intraspinal neoplasm; combined extradural-intradural lesion, any level	20.82%	1.20%
Group 3 Average			7.76%	0.41%
Group 4	63300	Vertebral corpectomy (vertebral body resection), partial or complete, for excision of intraspinal lesion, single segment; extradural, cervical	2.25%	0.10%
	63301	Vertebral corpectomy (vertebral body resection), partial or complete, for excision of intraspinal lesion, single segment; extradural, thoracic by thoracolumbar approach	18.90%	1.07%
	63303	Vertebral corpectomy (vertebral body resection), partial or complete, for excision of intraspinal lesion, single segment; extradural, lumbar or sacral by transperitoneal or retroperitoneal approach	4.53%	0.20%
Group 4 Average			8.56%	0.46%
Group 5	63304	Vertebral corpectomy (vertebral body resection), partial or complete, for excision of intraspinal lesion, single segment; intradural, cervical	33.89%	1.92%
	63305	Vertebral corpectomy (vertebral body resection), partial or complete, for excision of intraspinal lesion, single segment; intradural, thoracic by transthoracic approach	N/A	N/A
	63306	Vertebral corpectomy (vertebral body resection), partial or complete, for excision of intraspinal lesion, single segment; intradural, thoracic by thoracolumbar approach	N/A	N/A
	63307	Vertebral corpectomy (vertebral body resection), partial or complete, for excision of intraspinal lesion, single segment; intradural, lumbar or sacral by transperitoneal or retroperitoneal approach	N/A	N/A
Group 5 Average			N/A	N/A
Group 6	22100	Partial excision of posterior vertebral component (spinous process, lamina or facet) for intrinsic bony lesion, single vertebral segment; cervical	55.93%	2.72%
	22101	Partial excision of posterior vertebral component (spinous process, lamina or facet) for intrinsic bony lesion, single vertebral segment; thoracic	21.82%	1.24%
	22102	Partial excision of posterior vertebral component (spinous process, lamina or facet) for intrinsic bony lesion, single vertebral segment; lumbar	91.89%	4.18%
Group 6 Average			56.55%	2.71%
Group 7	22110	Partial excision of vertebral body, for intrinsic bony lesion, without decompression of spinal cord or nerve root(s), single vertebral segment; cervical	22.54%	1.28%
	22112	Partial excision of vertebral body, for intrinsic bony lesion, without decompression of spinal cord or nerve root(s), single vertebral segment; thoracic	14.55%	0.83%
	22114	Partial excision of vertebral body, for intrinsic bony lesion, without decompression of spinal cord or nerve root(s), single vertebral segment; lumbar	8.50%	0.48%
Group 7 Average			15.20%	0.86%
Total			12.68%	0.64%

**Table 3:** Total change and CAGR in inflation adjusted payments from 2000–2021

Grouping	CPT Code	Procedure Description	Total Change in Inflation Adjusted Payment (%)	CAGR
Group 1	63275	Laminectomy for biopsy/excision of intraspinal neoplasm; extradural, cervical	−35.90%	−2.10%
	63276	Laminectomy for biopsy/excision of intraspinal neoplasm; extradural, thoracic	−39.35%	−2.35%
	63277	Laminectomy for biopsy/excision of intraspinal neoplasm; extradural, lumbar	−49.88%	−3.24%
	63278	Laminectomy for biopsy/excision of intraspinal neoplasm; extradural, sacral	−34.92%	−2.02%
Group 1 Average			−40.01%	−2.43%
Group 2	63280	Laminectomy for biopsy/excision of intraspinal neoplasm; intradural, extramedullary, cervical	−31.07%	−1.76%
	63281	Laminectomy for biopsy/excision of intraspinal neoplasm; intradural, extramedullary, thoracic	−33.46%	−1.92%
	63282	Laminectomy for biopsy/excision of intraspinal neoplasm; intradural, extramedullary, lumbar	−35.31%	−2.05%
	63283	Laminectomy for biopsy/excision of intraspinal neoplasm; intradural, sacral	−48.76%	−3.13%
Group 2 Average			−37.15%	−2.22%
Group 3	63285	Laminectomy for biopsy/excision of intraspinal neoplasm; intradural, intramedullary, cervical	−33.79%	−2.11%
	63286	Laminectomy for biopsy/excision of intraspinal neoplasm; intradural, intramedullary, thoracic	−39.48%	−2.20%
	63287	Laminectomy for biopsy/excision of intraspinal neoplasm; intradural, intramedullary, thoracolumbar	−29.49%	−2.06%
	63290	Laminectomy for biopsy/excision of intraspinal neoplasm; combined extradural-intradural lesion, any level	−23.22%	−1.17%
Group 3 Average			−31.50%	−1.89%
Group 4	63300	Vertebral corpectomy (vertebral body resection), partial or complete, for excision of intraspinal lesion, single segment; extradural, cervical	−35.02%	−2.16%
	63301	Vertebral corpectomy (vertebral body resection), partial or complete, for excision of intraspinal lesion, single segment; extradural, thoracic by thoracolumbar approach	−24.44%	−1.38%
	63303	Vertebral corpectomy (vertebral body resection), partial or complete, for excision of intraspinal lesion, single segment; extradural, lumbar or sacral by transperitoneal or retroperitoneal approach	−33.57%	−2.00%
Group 4 Average			−31.01%	−1.85%
Group 5	63304	Vertebral corpectomy (vertebral body resection), partial or complete, for excision of intraspinal lesion, single segment; intradural, cervical	−14.92%	−0.84%
	63305	Vertebral corpectomy (vertebral body resection), partial or complete, for excision of intraspinal lesion, single segment; intradural, thoracic by transthoracic approach	N/A	N/A
	63306	Vertebral corpectomy (vertebral body resection), partial or complete, for excision of intraspinal lesion, single segment; intradural, thoracic by thoracolumbar approach	N/A	N/A
	63307	Vertebral corpectomy (vertebral body resection), partial or complete, for excision of intraspinal lesion, single segment; intradural, lumbar or sacral by transperitoneal or retroperitoneal approach	N/A	N/A
Group 5 Average			N/A	N/A
Group 6	22100	Partial excision of posterior vertebral component (spinous process, lamina or facet) for intrinsic bony lesion, single vertebral segment; cervical	−0.91%	−0.05%
	22101	Partial excision of posterior vertebral component (spinous process, lamina or facet) for intrinsic bony lesion, single vertebral segment; thoracic	−22.59%	−1.29%
	22102	Partial excision of posterior vertebral component (spinous process, lamina or facet) for intrinsic bony lesion, single vertebral segment; lumbar	21.94%	1.25%
Group 6 Average			−0.52%	−0.03%
Group 7	22110	Partial excision of vertebral body, for intrinsic bony lesion, without decompression of spinal cord or nerve root(s), single vertebral segment; cervical	−22.13%	−1.26%
	22112	Partial excision of vertebral body, for intrinsic bony lesion, without decompression of spinal cord or nerve root(s), single vertebral segment; thoracic	−27.21%	−1.56%
	22114	Partial excision of vertebral body, for intrinsic bony lesion, without decompression of spinal cord or nerve root(s), single vertebral segment; lumbar	−31.05%	−1.76%
Group 7 Average			−26.80%	−1.53%
Total			−28.39%	−1.69%

### Group 2- Laminectomy for intradural, extramedullary neoplasm (Fig. 2a, b and c)

**Fig. 2:**
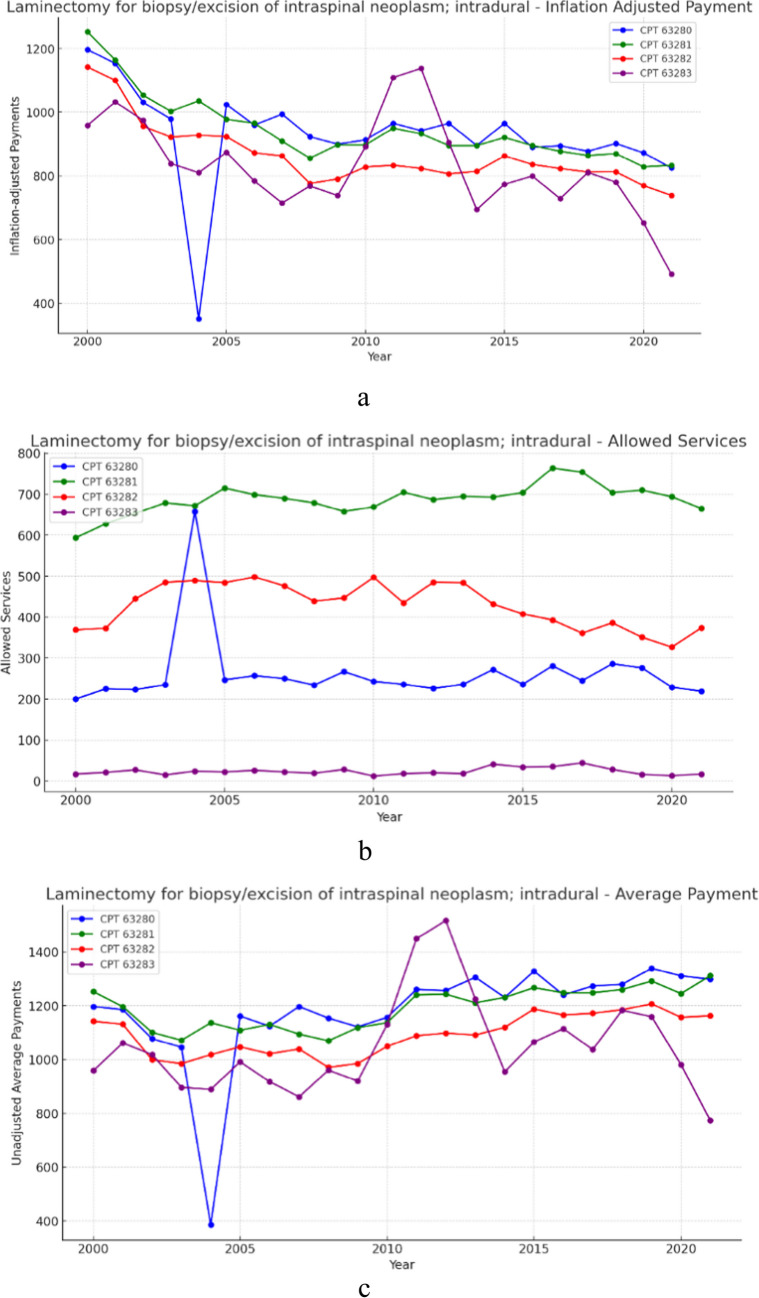
**a** 21 year, inflation adjusted payment trends for the selected procedure codes within laminectomy for biopsy/excision of intraspinal neoplasm; intradural. **b** 21 year, allowed services trends for the selected procedure codes within laminectomy for biopsy/excision of intraspinal neoplasm; intradural. **c** 21 year, unadjusted average payment trends for the selected procedure codes within laminectomy for biopsy/excision of intraspinal neoplasm; intradural

Over the 21-year period, allowed services for procedures within the intradural extramedullary group increased on average by 5.70% (Table 1). Meanwhile, unadjusted payments decreased on average by 1.09% (Table 2) and inflation adjusted average payment on average declined in real-value by 37.15% (Table 3). Results from the CAGR analysis indicate that, across this group, allowed services increased on average by 0.26% year-over-year (Table 1), unadjusted payments decreased on average 0.08% year-over-year (Table 2), and inflation adjusted payment on average fell 2.22% year-over-year (Table 3).

### Group 3- Laminectomy for intradural, intramedullary neoplasm + intradural/extradural (Fig. 3a, b and c)

**Fig. 3:**
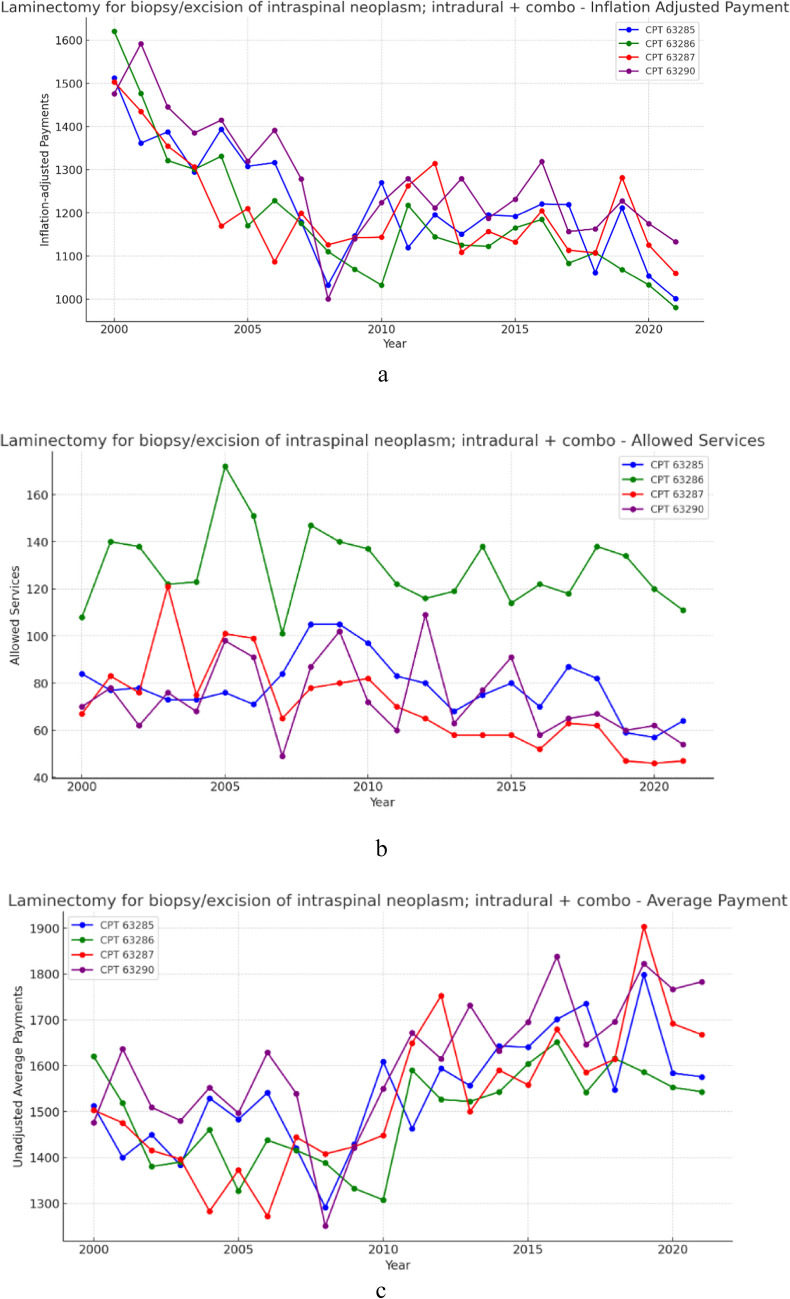
**a** 21 year, inflation adjusted payment trends for the selected procedure codes within laminectomy for intradural, intramedullary neoplasm + combination intradural/extradural. **b** 21 year, allowed services trends for the selected procedure codes within laminectomy for intradural, intramedullary neoplasm + combination intradural/extradural. **c** 21 year, unadjusted average payment trends for the selected procedure codes within laminectomy for intradural, intramedullary neoplasm + combination intradural/extradural

Over the 21-year period, allowed services for procedures within the intradural intramedullary group decreased on average 18.44% (Table 1). Meanwhile, unadjusted payments increased on average by 7.76% (Table 2) and inflation adjusted average payment on average declined in real-value by 31.50% (Table 3). Results from the CAGR analysis indicate that, across this group, allowed services decreased on average by 1.01% year-over-year (Table 1), unadjusted payments increased on average 0.41% year-over-year (Table 2), and inflation adjusted payment on average fell 1.89% year-over-year (Table 3).

### Group 4- Vertebral corpectomy for excision of intraspinal lesion – (extradural) (Fig. 4a, b and c)

**Fig. 4:**
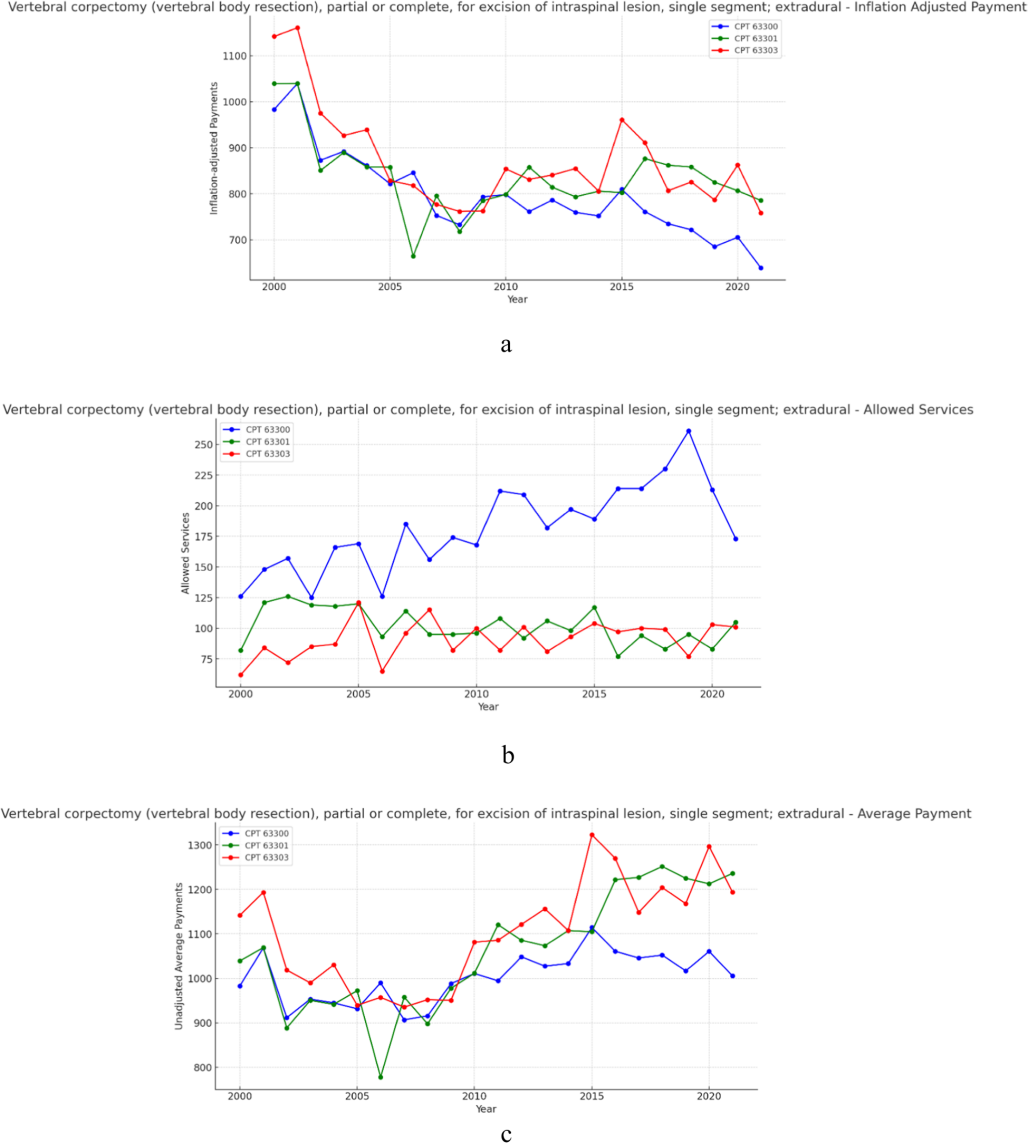
**a** 21 year, inflation adjusted payment trends for the selected procedure codes within vertebral corpectomy (vertebral body resection) for the excision of an intraspinal lesion (intradural, extramedullary). **b** 21 year, allowed services trends for the selected procedure codes within vertebral corpectomy (vertebral body resection) for the excision of an intraspinal lesion (intradural, extramedullary). **c** 21 year, unadjusted average payment trends for the selected procedure codes within vertebral corpectomy (vertebral body resection) for the excision of an intraspinal lesion (intradural, extramedullary)

Over the 21-year period, allowed services for procedures within the extradural vertebral corpectomy group increased on average 42.75% (Table 1). Meanwhile, unadjusted payments increased on average by 8.56% (Table 2) and inflation adjusted average payment on average declined in real-value by 31.01% (Table 3). Results from the CAGR analysis indicate that, across this group, allowed services increased on average by 2.14% year-over-year (Table 1), unadjusted payments increased on average 0.46% year-over-year (Table 2), and inflation adjusted payment on average fell 1.85% year-over-year (Table 3).

### Group 5- Vertebral corpectomy for excision of intraspinal lesion – (intradural) (Fig. 5a, b and c)

**Fig. 5:**
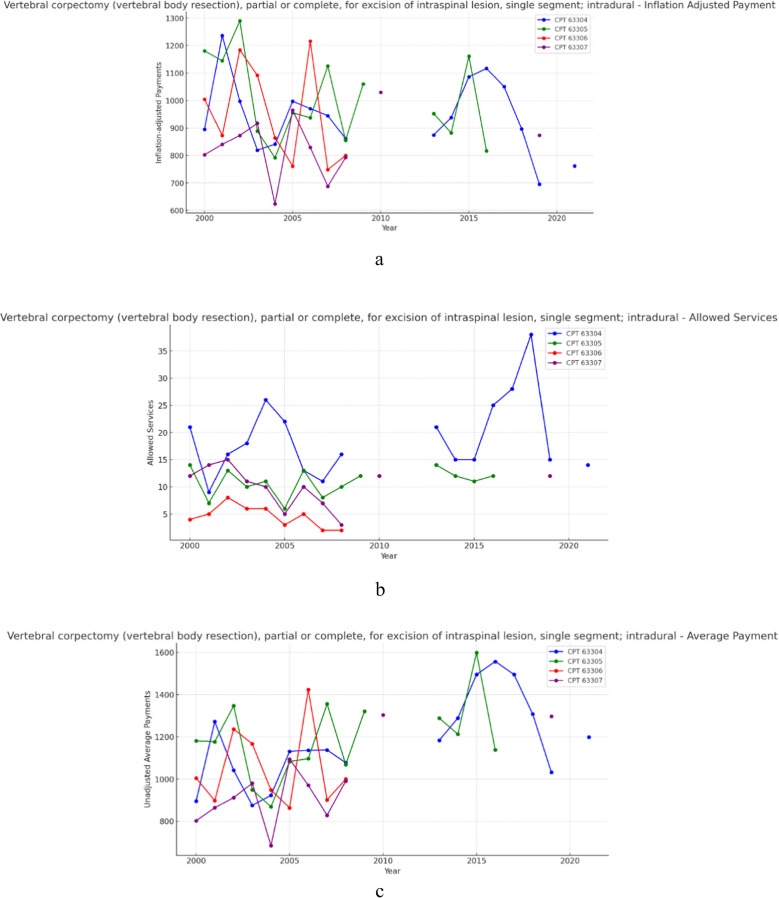
**a** 21 year, inflation adjusted payment trends for the selected procedure codes within vertebral corpectomy for excision of intraspinal lesion – (intradural, intramedullary). **b** 21 year, allowed services trends for the selected procedure codes within vertebral corpectomy for excision of intraspinal lesion – (intradural, intramedullary). **c** 21 year, unadjusted average payment trends for the selected procedure codes within vertebral corpectomy for excision of intraspinal lesion – (intradural, intramedullary)

Data analysis in this group was hampered by a lack of data points beyond 2008. Thus, this data was excluded from overall calculations. However, over the 8-year period (2000–2008) of intact data for all four included CPT codes, allowed services for procedures within the intradural vertebral corpectomy group decreased on average by 44.35%. Within the same timeframe, unadjusted payment increased on average by 8.45%. While average inflation adjusted payments declined on average by 13.24%. Results from the CAGR analysis for this group indicate that allowed services decreased on average by 5.54% year-over-year, while average unadjusted payments increased 1.06% year-over-year, and inflation adjusted payments fell on average by 1.66% year-over-year.

### Group 6—Partial excision of posterior vertebral component for intrinsic bony lesion (Fig. 6a, b, c and d)

**Fig. 6:**
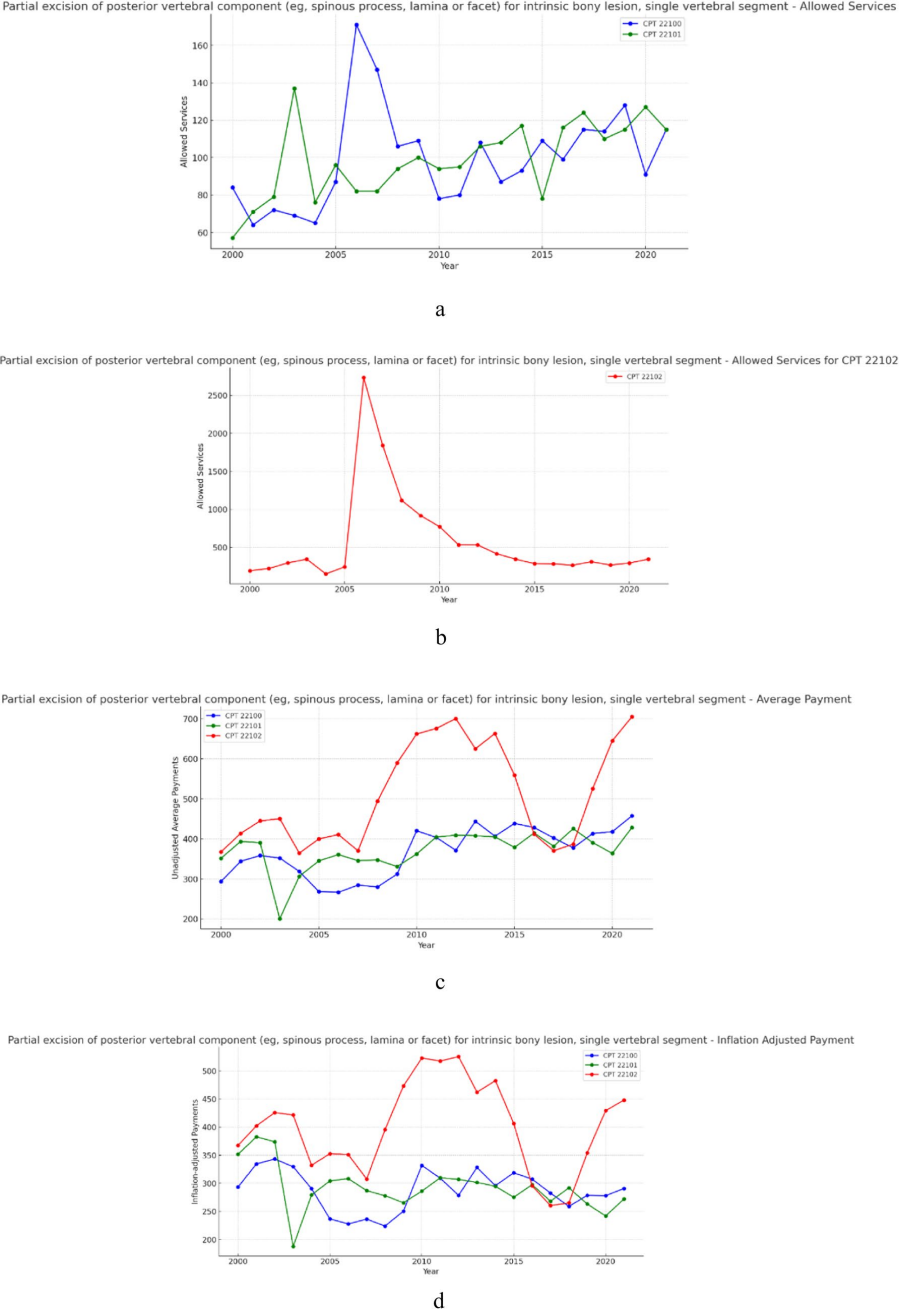
**a** 21 year, allowed services for the selected procedure codes within partial excision of posterior vertebral component for intrinsic bony lesion (excluding cpt 22102). **b** 21 year, allowed services for the selected procedure codes within partial excision of posterior vertebral component for intrinsic bony lesion (CPT 22102 only). **c** 21 year, unadjusted average payments for the selected procedure codes within partial excision of posterior vertebral component for intrinsic bony lesion. **d** 21 year, inflation adjusted average payments for the selected procedure codes within partial excision of posterior vertebral component for intrinsic bony lesion

Over the 21-year period, allowed services for procedures within the partial excision for posterior vertebral component for intrinsic bony lesion group increased on average by 72.47% (Table 1). Meanwhile, unadjusted payments increased on average by 56.55% (Table 2) and inflation adjusted average payment on average declined in real-value by 0.52% (Table 3). Results from the CAGR analysis indicate that, across this group, allowed services increased on average by 3.46% year-over-year (Table 1), unadjusted payments increased on average 2.71% year-over-year (Table 2), and inflation adjusted payments on average fell 0.03% year-over-year (Table 3).

### Group 7- Partial excision of vertebral body for intrinsic bony lesion (Fig. 7a, b and c)

**Fig. 7:**
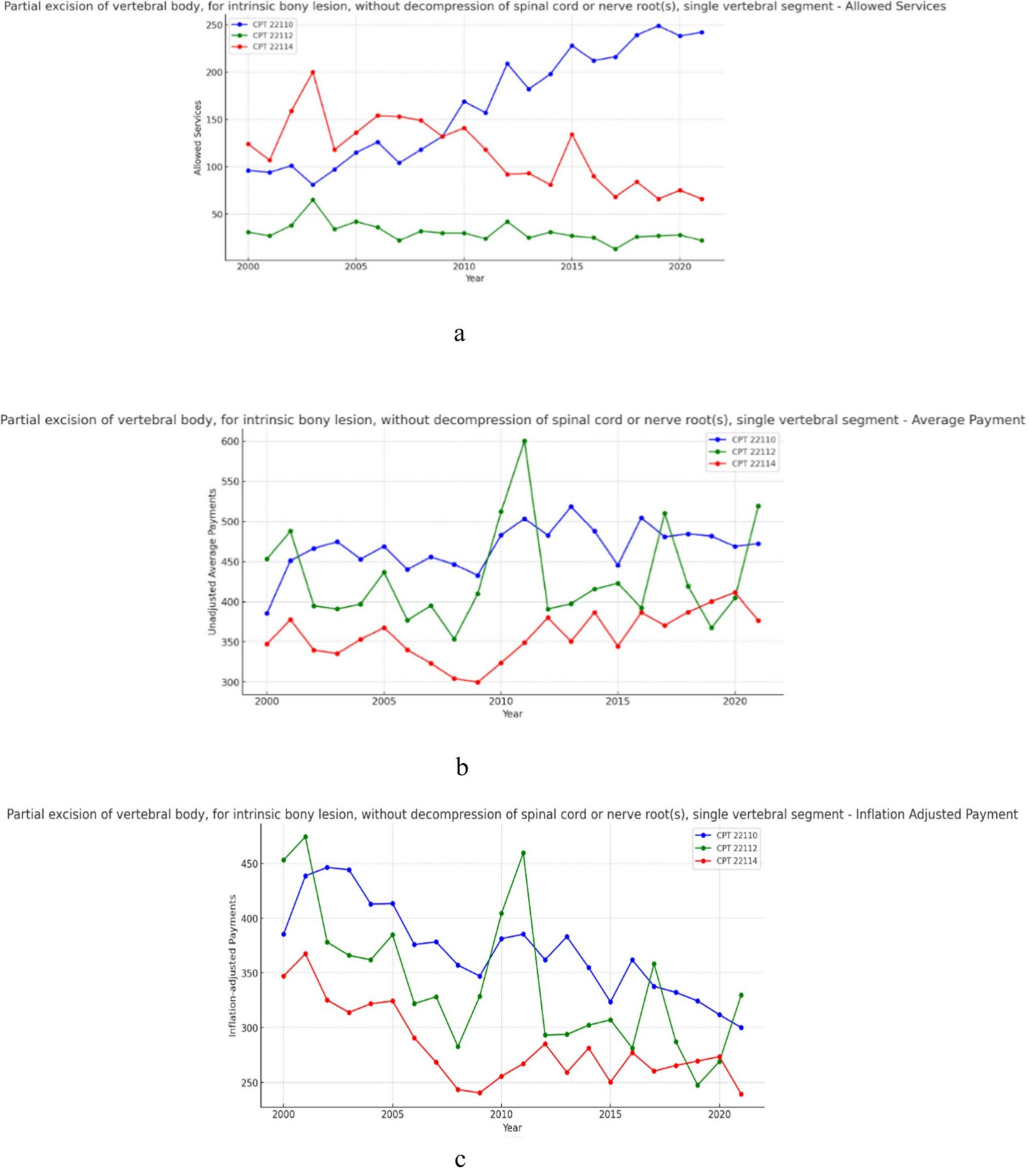
**a** 21 year, allowed services for the selected procedure codes within partial excision of vertebral body for intrinsic bony lesion. **b** 21 year, average payments for the selected procedure codes within partial excision of vertebral body for intrinsic bony lesion. **c** 21 year, inflation adjusted average payments for the selected procedure codes within partial excision of vertebral body for intrinsic bony lesion.

Over the 21-year period, allowed services for procedures within the partial excision for posterior vertebral body for intrinsic bony lesion group increased on average by 25.43% (Table 1). Meanwhile, unadjusted payments increased on average by 15.20% (Table 2) and inflation adjusted average payment on average declined in real-value by 26.80% (Table 3). Results from the CAGR analysis indicate that, across this group, allowed services increased on average by 0.85% year-over-year (Table 1), unadjusted payments increased on average 0.86% year-over-year (Table 2), and inflation adjusted payments on average fell 1.53% year-over-year (Table 3).

### General trends in patient payments

Over the 21-year period, total nominal patient payments across all groups rose on average by 15.20%. However, when adjusted for inflation, this represented an overall average decline of 26.80% in total real-value payment across all groups. The CAGR analysis revealed that allowed services across all groups increased on average by 0.85% per year, matching the average annual increase of 0.86% in unadjusted payments. Yet when adjusted for inflation, this resulted in an average annual decrease of 1.53% in real-value payment across all groups.

### Noteworthy individual and overall findings

Moreover, several individual CPT codes within the scope of the present study are noteworthy. CPT codes 63304 and 63290 (“cervical corpectomy; intradural” and “any level laminectomy; combined intradural/extradural”) saw their unadjusted total payment rates rise by 33.89% and 20.82% respectively, yet each ended the period yielding negative total inflation adjusted real-value returns (14.92% and 23.22%). The reimbursement rate that was most eroded by inflation was that of CPT code 63283 (sacral laminectomy; intradural), which saw a decreased total nominal return rate of 19.36% and a drop in adjusted total real-value return rate of 48.76%.

**Fig. 8:**
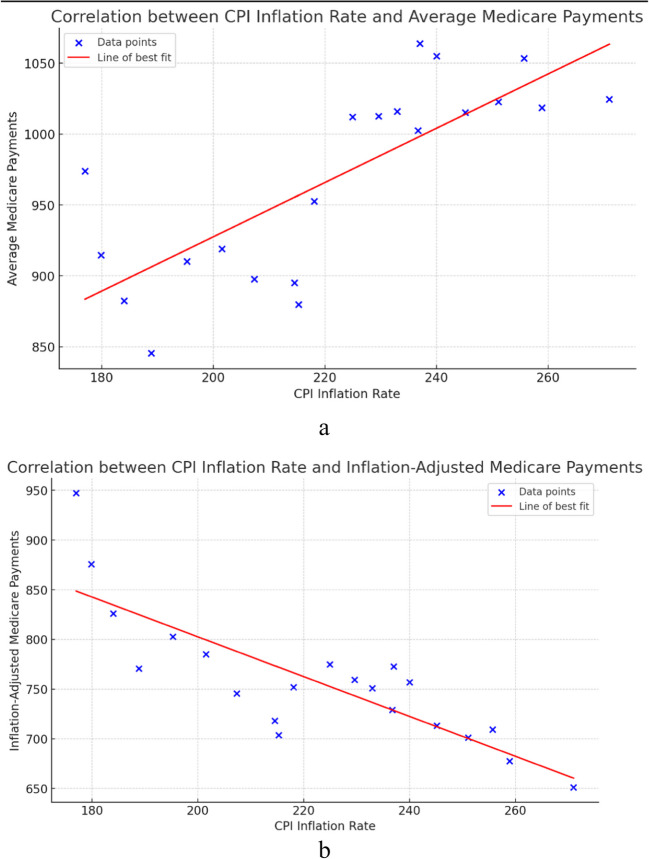
**a** Scatter plot showing the relationship between CPI and nominal Medicare payments, with a line of best fit indicating a strong positive correlation. **b** Scatter plot showing the relationship between CPI and inflation-adjusted Medicare payments, with a line of best fit indicating a strong negative correlation

The Pearson correlation coefficient between the CPI and the nominal Medicare payments was r = 0.775 (*p* < 0.001) This indicates a strong positive correlation between the CPI and nominal Medicare payments over the study period. (Fig. 8a) The Pearson correlation coefficient between the CPI and the inflation-adjusted Medicare payments was *r* = 0.824 (*p* < 0.001). This indicates a strong negative correlation between the CPI and inflation adjusted Medicare payments. (Fig. 8b).

## Discussion

Across all categories observed, unadjusted reimbursements have remained stable to slightly increased. However, there is a trend of decreasing real value in medical service payments when adjusted relative to CPI inflation statistics over the 21-year period. Applying this adjustment to the Medicare Part B payment schedule reveals the following two concerns: first, real generalized inflation appears to have outpaced physician reimbursement inflation. Secondly, the decreasing real value of reimbursement rates has not kept pace with the rising costs of tumor resections, creating a significant cost discrepancy. Interestingly, data released by the BLS indicate the CPI medical care index approximately doubled from 2000–2021 [14]. Meanwhile, this acceleration exceeds the general CPI data suggesting the U.S. dollar has lost approximately 33% of its purchasing power throughout the same timeframe [15]. Despite these figures, the data presently furnished suggest reimbursements for surgeons performing spinal tumor resections has suffered. To better contextualize this discussion, note that a 2016 study by Lau et. al which found that the mean direct cost of hospital admission for the surgical management of spinal tumors was $52,083 [16].

The data highlighted a decline in reimbursement rates for procedures with the most allowed services by Medicare. Within the subcategory of extradural resections, CPT 63276 (thoracic laminectomy; extradural) remained the most in-demand service while also accumulating the greatest relative increase in allowed services. The second most utilized service in this subcategory, CPT 63277 (lumbar laminectomy; extradural), showed a stable number of services allowed. However, both of these extradural surgeries saw decreases in inflation-adjusted reimbursement of 2.35% and 3.24% respectively. Moreover, CPT 63277 received the lowest absolute inflation-adjusted payment rate in this subcategory despite its relative frequency.

The subcategory of intradural, extramedullary tumor resection displays stable trends in allowed services across the twenty-one-year span and decreasing inflation-adjusted payments for all procedures. This data elicits two noteworthy observations regarding the influence of demand on reimbursement for CPT codes 63280 and 63283 (“cervical laminectomy; intradural, extramedullary” and “sacral laminectomy; intradural”). The year 2004 saw an impressive spike in allowed services for CPT 63280, which precipitated an antithetical nadir in real value payments for that procedure. Both allowed services and inflation-adjusted payments for this surgery returned to their prior trajectories immediately following 2004 with no apparent propagating effects within its subcategory. Here, it simply appears Medicare did not accomplish price adjustments reflective of real-time market dynamics. For CPT 63283, the studied time-frame saw stable, low-level utilization of this billing code. Despite its relatively low employment, CPT 63283 saw a sudden rise in inflation-adjusted payments from 2009–2012. Between 2011–2012, despite no concomitant rise in utilization, CPT 63283 offered the greatest real-value payment within this subcategory and a relatively high yielding service until 2014. Again, this data suggests a misalignment of the pricing mechanism, which if further exacerbated, may have potential consequences on innovation and advancements in quality of patient care within this subset of spinal procedures.

The subcategory representing partial excision of posterior vertebral elements offers findings tangential to this point. Allowed services for CPT 22102 (lumbar partial posterior excision; bony) saw a significant rise in 2006, which then tapered down towards its baseline employment by around 2011, while the two other CPTs within this subcategory- 22100 (cervical partial posterior excision; bony) and 22101 (thoracic partial posterior excision; bony)- also display employment spikes around this time. However, the rise in allowed services for CPT 22101 had no obvious correlation to payments. Interestingly, the steep rise in utilization of CPT 22102 seems to have provoked a subsequent rise in real-value reimbursements, which peaked in 2010–2012, several years after the dramatic 2006 utilization spike and during a time when its employment was reverting back to baseline. This procedure is also noteworthy due to its large service volume. It may be the case that this extent of service volume enabled a larger number of surgeons to unify into a more perceptible entity better capable of appealing to policymakers responsible for drafting Medicare payment schedules.

Our analysis revealed a paradox wherein patient out-of-pocket expenses, encapsulated by the CPI-MEDSL, notably doubled within the study period. In contrast, Medicare reimbursements for spinal tumor resection procedures, when adjusted for inflation, declined over this period. This discrepancy highlights a growing financial burden on patients, which is not mirrored in the reimbursement patterns for these procedures. Despite escalating costs borne by patients, indicative of heightened medical care valuations from a consumer standpoint, procedural reimbursement rates do not reflect adjustments for inflation, suggesting a devaluation of spinal tumor surgeries over time. The factors contributing to this divergence are complex and warrant further exploration.

A limitation of this study is the reliance solely on reimbursement rate Medicare part B data, which may introduce bias as this dataset is limited to patients 65 years or older. This may not fully represent the entire spectrum of the spinal surgery market which could include patients younger than 65 years old, thus it is unclear if payments are reflective of the incidence of procedures in the younger population. Additionally, this data does not account for reimbursement of coupled/associated procedures, for example, the common occurrence of fusion procedures alongside neoplasm excisions. Because a similar phenomenon has been shown to affect these concomitant procedures [4, 5], complex cases involving both tumor resection and fusion are subject to compounded devaluations such that our current results likely underestimate inflationary erosion in certain circumstances. However, in some instances it may be possible that increases in associated procedures could impact perceived reimbursement rates for excisions performed within complex cases if excision codes have been artificially lowered to account for expensive predicted associated instrumentation coding. The absence of comparative data from private or third-party insurers also limits the generalizability of the findings, as reimbursement dynamics can significantly vary across different payers. Additionally, the study did not account for geographic factors accounted for by the geographic practice cost index (GPCI), which can play a crucial role in influencing reimbursement rates and patient access to specialized care. Careful evaluation of geographic reimbursement trends may be of value in the future. Furthermore, the study's timeframe, concluding in 2021, may not capture the complete ramifications of the Coronavirus pandemic and the subsequent shifts in the economic landscape, necessitating cautious interpretation of the findings in the context of a rapidly changing healthcare environment. However, we would expect the pandemic’s impact on the trends of spinal tumor reimbursements to be self-limited to several years rather than permanent.

To this point, economic downturns, such as the 2008 financial crisis and subsequent recessions, may have influenced health care spending, reimbursement structures, and patient access to specialized procedures. These fluctuations could introduce confounding variables that are not explicitly addressed in the analysis. The study's focus on reimbursement rates may not fully capture the complex interplay between economic downturns and the financial dynamics of subspecialty spinal procedures.

Another factor that can aid in explaining the decreasing adjusted reimbursement in neurosurgery demonstrated in this study is through underlying congressional policy during this time period. From 1997 to 2015, CMS determined Medicare and Medicaid reimbursement utilizing the sustainable growth rate (SGR) under the Balanced Budget Act of 1997. This legislation was originally proposed to control Medicare spending on physician reimbursement in an effort to balance the United States federal budget by 2002 [4]. The SGR was set annually and determined changes to Medicare reimbursement for procedures based on the prior year's data. This reactive approach led to a lag in adjusting reimbursement rates to keep up with rising healthcare costs, contributing to the decreasing trends observed in reimbursement rates. Additionally, a bipartisan congress vote to implement the Medicare Access and CHIP Reauthorization Act (MACRA), has significantly impacted physician reimbursement rates within our study. The introduction of MACRA in 2015 replaced the sustainable growth rate (SGR) formula with two new payment tracks: the Merit-based Incentive Payment System (MIPS) and Advanced Alternative Payment Models (APMs). While MACRA aimed to reward value and quality of care, the shift to these new models brought unintentional strain and complexity. The combined effect of reduced Medicare reimbursements and the transition to MACRA's value-based payment models has pressured physicians to adapt to new financial realities, often focusing changes in practice management and patient care strategies. These changes have created a challenging environment for many providers, impacting total reimbursement rates.

Therefore, it is crucial to recognize that the observed trends may be influenced by broader economic factors and policy changes and the study's conclusions should be interpreted within the context of the specific economic conditions during the analyzed period. These limitations underscore the importance of interpreting the study results within the specified scope and acknowledging the need for future research endeavors to address these constraints.

## Conclusion

General trends show a decline in reimbursement rates for spinal tumor resection between 2000 and 2021. This decline poses significant challenges to healthcare providers and signals the need for a comprehensive reevaluation of reimbursement structures. Moving forward, it will be necessary for collaborative efforts to be undertaken by healthcare professionals, policymakers, and industry leaders to advocate for compensation reflective of the assumed risks, necessary skills, and extensive training requisite of the associated procedures. Stakeholders can, and should, work proactively towards ensuring the sustainability of specialized spine care, thereby continuing the effort to optimize structures and promote development of patient care in the evolving field of spinal surgery.

## Data Availability

All raw Medicare part B data was retrieved from data.cms.gov.

## References

[1] Jen MY, Han V, Bennett K et al (2020) Public performance metrics: driving physician motivation and performance. West J Emerg Med 21(2):247–251. 10.5811/westjem.2020.1.41798 32191182 10.5811/westjem.2020.1.41798 PMC7081853

[2] Petersen LA, Simpson K, Pietz K et al (2013) Effects of individual physician-level and practice-level financial incentives on hypertension care: a randomized trial. JAMA 310(10):1042–1050. 10.1001/jama.2013.276303 24026599 10.1001/jama.2013.276303 PMC4165573

[3] Sutton M, Nikolova S, Boaden R, Lester H, McDonald R, Roland M (2012) Reduced mortality with hospital pay for performance in England. N Engl J Med 367(19):1821–1828. 10.1056/NEJMsa1114951 23134382 10.1056/NEJMsa1114951

[4] Haglin JM, Zabat MA, Richter KR et al (2022) Over 20 years of declining Medicare reimbursement for spine surgeons: a temporal and geographic analysis from 2000 to 2021. J Neurosurg Spine. 10.3171/2022.2.SPINE211368 35334463 10.3171/2022.2.SPINE211368

[5] Honarpisheh P, Parker SL, Conner CR et al (2024) 20-year inflation-adjusted medicare reimbursements (Years: 2000–2020) for common lumbar and cervical degenerative disc disease procedures. Global Spine J 14(1):211–218. 10.1177/21925682221100173 35609345 10.1177/21925682221100173 PMC10676153

[6] U.S. Personal Health Care Spending By Age and Sex 2020 Highlights. Centers for Medicare and Medicaid Services. chrome-extension://efaidnbmnnnibpcajpcglclefindmkaj/https://www.cms.gov/Research-Statistics-Data-and-Systems/Statistics-Trends-and-Reports/NationalHealthExpendData/Downloads/AgeandGenderHighlights.pdf. Accessed 31 Jan 2024

[7] Rychen J, Stricker S, Mariani L, Schaeren S, Jost GF (2019) Outcome of spinal surgery in patients older than age 90 years. World Neurosurg 123:e457–e464. 10.1016/j.wneu.2018.11.188 30500575 10.1016/j.wneu.2018.11.188

[8] López-Otín C, Blasco MA, Partridge L, Serrano M, Kroemer G (2013) The hallmarks of aging. Cell 153(6):1194–1217. 10.1016/j.cell.2013.05.039 23746838 10.1016/j.cell.2013.05.039 PMC3836174

[9] Lodato MA, Rodin RE, Bohrson CL et al (2018) Aging and neurodegeneration are associated with increased mutations in single human neurons. Science 359(6375):555–559. 10.1126/science.aao4426 29217584 10.1126/science.aao4426 PMC5831169

[10] Teo HE, Peh WC (2004) Primary bone tumors of adulthood. Cancer Imaging 4(2):74–83. 10.1102/1470-7330.2004.0004. (Published 2004 Mar 29) 18250012 10.1102/1470-7330.2004.0004 PMC1434587

[11] Maynou L, Pearson G, McGuire A, Serra-Sastre V (2022) The diffusion of robotic surgery: examining technology use in the English NHS. Health Policy 126(4):325–336. 10.1016/j.healthpol.2022.02.007 35307200 10.1016/j.healthpol.2022.02.007

[12] D’Souza M, Gendreau J, Feng A, Kim LH, Ho AL, Veeravagu A (2019) Robotic-assisted spine surgery: history, efficacy, cost, and future trends [published correction appears in Robot Surg. 2019 Dec 23;6:25]. Robot Surg 6:9–23. 10.2147/RSRR.S190720. (Published 2019 Nov 7) 31807602 10.2147/RSRR.S190720 PMC6844237

[13] “Consumer Price Index for All Urban Consumers: Medical Care in U.S. City Average.” FRED, 12 June 2024, fred.stlouisfed.org/series/CPIMEDSL. Accessed 08 July 2024

[14] Databases, Tables & Calculators by Subject. Bureau of Labor Statistics. https://data.bls.gov/pdq/SurveyOutputServlet. Accessed 1 Feb 2024

[15] CPI Inflation Calculator. Bureau of Labor Statistics. https://www.bls.gov/data/inflation_calculator.htm. Accessed 1 Feb 2024

[16] Lau D, Chan AK, Theologis AA, Chou D, Mummaneni PV, Burch S, Berven S, Deviren V, Ames C (2016) Costs and readmission rates for the resection of primary and metastatic spinal tumors: a comparative analysis of 181 patients. J Neurosurg Spine 25(3):366–378. 10.3171/2016.2.SPINE15954 27129043 10.3171/2016.2.SPINE15954

